# How active are women who play bingo: a cross-sectional study from the Well!Bingo project

**DOI:** 10.1186/s12905-017-0405-z

**Published:** 2017-07-28

**Authors:** Gemma C. Ryde, Trish Gorely, Ruth Jepson, Cindy Gray, Ashley Shepherd, Dionne Mackison, Aileen V. Ireland, Brian Williams, Marion E. T. McMurdo, Josie M. M. Evans

**Affiliations:** 1 Faculty of Health Sciences and Sport, University of Stirling, Scotland, FK9 4LA UK; 2 Scottish Collaboration for Public Health Research and Policy, 20 West Richmond St, Edinburgh, EH8 9DX UK; 3 Institute of Health and Wellbeing, College of Social Sciences, University of Glasgow, G12 8RS, Glasgow, UK; 4 NHS Health Scotland, Edinburgh, UK; 5 Medical Research Institute, University of Dundee, Ninewells Hospital, Dundee, DD1 9SY UK

**Keywords:** Physical activity, Sedentary behaviour, Accelerometer, Community, Health

## Abstract

**Background:**

The benefits of physical activity are well established, yet large numbers of people are not sufficiently active to gain health benefits. Certain population groups are less physically active than others, including older women from areas of high economic deprivation. The Well!Bingo project was established with the aim of engaging such women in the development of a health promotion intervention in a bingo club. This paper reports on the assessment of health status, physical activity and sedentary behaviour of women attending a bingo club in central Scotland, UK as part of the Well!Bingo project.

**Methods:**

Women attending the bingo club were invited to provide information on demographic characteristics, and self-reported physical activity and sedentary behaviour via a self-complete questionnaire as part of a cross-sectional study (*n =* 151). A sub-sample (*n* = 29) wore an accelerometer for an average of 5.7 ± 1.4 days. Differences between younger (under 60 years) and older adults (60 years and over) were assessed using a chi-square test for categorical data and the independent samples *t*-test was used to assess continuous data (*p* < 0.05).

**Results:**

The mean age was 56.5 ± 17.7 years, with 57% living in areas of high deprivation (Scottish Index of Multiple Deprivation quintile one and two). Sixty-three percent of women (*n* = 87) reported they were meeting physical activity guidelines. However, objective accelerometer data showed that, on average, only 18.1 ± 17.3 min a day were spent in moderate to vigorous physical activity. Most accelerometer wear time was spent sedentary (9.6 ± 1.7 h). For both self-report and accelerometer data, older women were significantly less active and more sedentary than younger women. On average, older women spent 1.8 h more than younger women in sedentary activities per day, and took part in 21 min less moderate to vigorous physical activity (9.4 mins per day).

**Conclusion:**

The findings of this study suggest that bingo clubs are settings that attract women from areas of high deprivation and older women in bingo clubs in particular would benefit from interventions to target their physical activity and sedentary behaviour. Bingo clubs may therefore be potential intervention settings in which to influence these behaviours.

## Background

The association between physical activity and health is well established. Physical activity is beneficial for the prevention of conditions such as cardiovascular disease, mental health, obesity and diabetes and also the treatment of these conditions [1–3]. In both developed and non-developed countries, population levels of physical activity are low, with large numbers of people not sufficiently active to gain health benefits [4]. As well as having detrimental effects on an individual’s health, physical inactivity also has significant financial implications on healthcare systems. It has been estimated that inactivity costs the UK economy alone 9 billion pounds per year, with similar figures reported in Australia, the US and Canada [5, 6].

Certain demographic and socio-economic groups within the population are less physically active than others. For example, in Scotland, women are less likely to meet the weekly recommended physical activity guidelines than men (58 and 67%, respectively) [7]. Older women are also less active than younger women, with adherence to guidelines in those aged 75 and over almost half that of those aged 55 to 74 [7]. Social and economic disparities in physical activity levels in Scotland may also exist for women, with a 14% difference in the proportion of women meeting guidelines from the most deprived areas than those in the least deprived areas [8]. Women with lower educational attainment are also less active than those with higher attainment [9]. Combined, these results suggest a possible gradient in activity levels by socio-economic status. Despite the need for increased levels of physical activity in these populations, it is often difficult to identify, target and engage with these groups.

Bingo clubs are a setting that could offer a potential avenue to engage these target groups in physical activity behaviour change. The Well!Bingo project was established in March 2013 with the aim of using community-based participatory research to engage women in the development of a health promotion intervention in a bingo club in Central Scotland. Prior to the development of an intervention, cross-sectional data were collected to: 1) describe the socio-demographic characteristics of bingo players at the club; and 2) to assess their health status, physical activity and sedentary behaviour. This paper reports on these cross-sectional data.

## Methods

### Study design and participants

Members of a bingo club in central Scotland were invited to participate in this cross-sectional study. The study had two components: (1) all participants completed a questionnaire; and (2) a subsample wore accelerometers and completed a wear time diary. Participants were recruited through an information stall set up by the research team in the foyer at the only entrance to the bingo club. The stall was manned by at least two members of the research team on the busiest morning, daytime and evening bingo sessions over a 2-week period, including weekends. Announcements were made by the bingo club staff before and after bingo sessions to encourage people to complete the questionnaire. Completed questionnaires were returned to researchers or deposited in a sealed return box when the stall was unmanned.

As an incentive, all participants who completed the questionnaire were entered into a prize draw with the chance of winning a gift voucher worth £100. Questionnaires were completed anonymously, and consent was implied through return of the questionnaire. Contact details were collected and kept separately for entry into the prize draw, and respondents could indicate whether they would be interested in wearing an accelerometer. Those who registered interest in wearing an accelerometer were contacted by telephone, and researchers arranged to meet them individually at the bingo club to collect informed consent and distribute the accelerometer and wear time diary. Accelerometers were then collected in person at the bingo club by prior arrangement after at least 7 days had passed. The study was approved by the School of Nursing, Midwifery and Health Research Ethics Committee of the University of Stirling, Scotland (REF: SREC 13/14 Paper No.80).

### Socio-demographic characteristics

Participants were asked to state their age, gender, postcode, ethnic background, highest level of educational qualification, whether they lived alone, marital status, self-reported health status and self-reported ill health. For self-reported ill health, a free text option allowed the specification of health conditions. Postcode was used as a proxy group measure of material deprivation using the Scottish Index of Multiple Deprivation (SIMD) [10]. The SIMD assesses levels of income, employment, health, education, geographic access, housing and crime in postcode areas, which are then categorised into quintiles from the highest areas of deprivation (SIMD 1) to the lowest (SIMD 5).

### Self-report physical activity and sitting time

Self-reported physical activity was measured using the Active Australia Questionnaire [11, 12]. Questions were asked relating to frequency (number of times) and duration (hours and minutes) of activities during the previous week, including vigorous activities (jogging, cycling, aerobics, competitive tennis), moderate activities (gentle swimming, social tennis, golf), and walking for recreation (>10 min bouts of walking for exercise or to get to or from places). Data were cleaned following published recommendations for the analysis and reporting of physical activity data [11, 12]. Mean number of minutes spent walking, and in moderate or vigorous physical activity, was calculated. Total physical activity was calculated as the sum of these three categories. These data were used to identify participants who met physical activity guidelines. This was defined as accumulating a total of 150 min of activity (walking, moderate and vigorous) in the previous week, irrespective of frequency of activities. Frequency was excluded to reflect the current approach taken for the calculation of the UK national data.

Sitting time was measured using a previously validated domain-specific question [13]. Five domains were used to assess how much time people sat (hours and minutes) on a weekday and a weekend day. Domains included sitting while travelling to and from places, at work, watching TV, home computer use, and in leisure time excluding TV (visiting friends, movies, dining out). Mean sitting time per domain on both a weekday and weekend was calculated. Time reported in each domain was summed to create an estimate of total weekday and total weekend sitting.

### Objective physical activity, sedentary behaviour and diary data

Accelerometers (ActiGraph GT3X+) were distributed through a brief (15 min) one-to-one session with each participant. Accelerometers were initialised to record data at 30 Hz. Participants were asked to wear the accelerometer around the waist for 7 days during all waking hours, except when showering, bathing or swimming. Diaries were provided to record time of waking, when they went to bed, non-wear time and when they attended the bingo. Accelerometer data were downloaded using ActiLife software and saved in 60-s epochs (version 6.9.0; Full Edition). Data were also cleaned in the ActiLife software using a validated algorithm to identify non-wear time (continuous zeros for 90 min, spike tolerance 2 min) [14]. Non-wear time data were cross-checked with diary entries, and sedentary time accumulated whilst wearing the accelerometer and sitting playing bingo that was classified as non-wear time by the accelerometer was recoded as wear time and included in the analysis. Data were included if accelerometer wear time totalled at least 10 h per day, with a minimum of 3 days of data [15]. The number of days with >10 h of data (number of valid wear days), and the average number of hours wearing the device per day (wear time per day), were calculated. Data were then sorted into activity categories using established uni-axial-based thresholds as follows: sedentary activity (99 counts), light activity (100 to 2019 counts) and moderate to vigorous physical activity (>2020 counts) [15]. The percentage of wear time and the time per day spent in each activity category were calculated.

### Data management and analysis

All data were analysed using SPSS (IBM SPSS Statistics v21.0.0). Categorical variables were presented as number and proportions and continuous variables as means ± standard deviations (SD). Results are presented for the total sample, categorised into those aged under 60 years (referred to as younger) and those aged 60 years and over (referred to as older). Older adults were defined based on the United Nations’ agreed cut-offs and the earliest pension age in the UK [16]. Differences between younger and older participants were assessed using chi-square for categorical data. Where sample size assumptions for chi-squared were violated (more than 20% of cells with expected counts of less than 5), categories were combined. The independent samples *t*-test was used to assess continuous data (*p* < 0.05).

## Results

### Sample

Weekly attendance (numbers of membership cards swiped) at the club during the recruitment period was approximately 1200. This included some repeat visits by individual members, i.e. the same member attending more than once a week. The questionnaire was returned by 151 women. There were responses from 11 men, but these were excluded from further analysis as the aim of the Well!Bingo project was to target women. Interest in wearing an accelerometer was registered by 77 women. Thirty-three wore an accelerometer with four excluded from analysis (1 pregnant, 1 lost the accelerometer, 2 had insufficient data), leaving 29 in the final sample.

### Socio-demographic information

Socio-demographic information for the whole sample and for younger and older women are shown in Table 1. The mean age was 56.5 ± 17.7 years (range 18 to 91 years), with 48% aged 60 years and over (*n* = 69). Most women lived in areas of higher deprivation (57%, SIMD 1 and 2) with only 4% (*n* = 5) living in the least deprived areas (SIMD 5). There was limited ethnic diversity, with 97% of respondents (*n* = 141) reporting their background as white Scottish. Most respondents had no formal qualification (53%, *n* = 72), were married or cohabiting (50%, *n* = 73) and did not live alone (68%, *n* = 99). Having ill health was reported by 53 women (39%), with 27 reporting having two or more conditions that adversely impacted their health. Forty-seven women reported the specified health condition, with the most common being arthritis and joint problems (*n* = 20); heart and respiratory conditions (*n* = 12) (including angina, heart attack, stroke and chronic obstructive pulmonary disease); and diabetes (*n* = 9). Self-reported health status was mostly rated as good or very good/excellent (69%). Between age groups, there were no differences reported for SIMD or ethnic background, health status or ill health. Older women were more likely than younger women to have no formal educational qualification (*p* = 0.027), live alone (*p* = 0.012), be single or never married (*p* < 0.000) and be retired (*p* < 0.000).

**Table 1:** Demographic and self-reported health data of female bingo players

Characteristics	Total	Under 60	60 and over	Difference between groups *p* < 0.05
Age years (*n* = 145) M ± SD	56.5 ± 17.7	43.2 ± 13.4	71.1 ± 7.1	NA
SIMD quintiles (*n* = 147) *n* (%)				
1 (0–20%) most deprived	35 (24)	22 (29)	13 (19)	
2 (20–40%)	49 (33)	27 (36)	20 (30)	
3 (40–60%)	27 (18)	11 (14)	16 (24)	
4 (60–80%)	31 (21)	13 (17)	16 (24)	
5 (80–100%) least deprived	5 (4)	3 (4)	2 (3)	
Ethnic background (*n* = 147) *n* (%)				
Other	5 (3)	1 (1)	4 (6)	
White Scottish	142 (97)	75 (99)	64 (94)	
Qualification (*n* = 136) *n* (%)				
University or higher	10 (7)	9 (13)	1 (2)	
Certificate/diploma/trade	54 (40)	29 (41)	25 (38)	
No formal qualification	72 (53)	32 (46)	39 (60)	*p* = 0.027
Employment status (*n* = 135) *n* (%)				
Employed	57 (42)	49 (68)	8 (13)	
Unemployed	29 (22)	22 (31)	7 (11)	
Retired	49 (36)	1 (1)	48 (76)	*P* < 0.001
Living alone (*n* = 145) *n* (%)				
No	99 (68)	58 (78)	40 (59)	*p* = 0.012
Yes	46 (32)	16 (22)	28 (41)	
Marital status (*n* = 143) *n* (%)				
Separated or divorced	24 (16)	12 (16)	10 (15)	
Widowed	29 (20)	3 (4)	24 (36)	*P* < 0.001
Single/never married	30(14)	19 (25)	1 (2)	
Married or cohabiting	73 (50)	41 (55)	31 (47)	
Self-reported health status (*n* = 143) *n* (%)				
Poor	14 (9)	6 (8)	8 (12)	
Fair	33 (22)	16 (21)	16 (24)	
Good	52 (35)	32 (42)	17 (25)	
Very good/excellent	50 (34)	22 (49)	26 (39)	
Self-reported ill health (*n* = 135) *n* (%)				
None	82 (61)	47 (66)	31 (53)	
Yes	53 (39)	24 (34)	28 (48)	

*NA* not applicable

### Self-reported physical activity and sitting time

The results for physical activity and sitting time of bingo players are shown in Table 2. For self-reported physical activity, 63% of women (*n* = 87) were found to be meeting physical activity guidelines, defined as 150 min of moderate to vigorous physical activity per week. Overall, a mean of 5.6 ± 5.3 h of physical activity per week was reported, with most (58%) spent walking. Significant differences between older and younger women were found for total physical activity (*p* = 0.024), and walking (*p* = 0.009), with older women accumulating less total physical activity and less walking per day than younger women. A higher percentage of younger than older women were found to be meeting physical activity guidelines (73% and 51% respectively; *p* = 0.009). No significant differences were found in self-reported moderate or vigorous physical activity time between younger and older women.

**Table 2:** Self-report and objective physical activity and sedentary time of female bingo players

Characteristics	Total	Under 60	60 and over	Difference between groups
Self-report data				
Physical activity time hrs (*n* = 139) M ± SD				
Total PA	5.6 ± 5.3	6.6 ± 5.4	4.4 ± 4.8	*t*(131) = −2.28, *p* = 0.024
Vigorous	0.9 ± 1.4	0.9 ± 1.5	0.7 ± 1.3	
Moderate	1.0 ± 2.0	1.0 ± 1.9	0.9 ± 1.8	
Walking	3.7 ± 4.0	4.6 ± 4.6	2.8 ± 2.9	*t*(131) = −2.65, *p* = 0.009
Meeting 150mins PA guidelines (*n* = 139) n (%)				
Meeting guidelines	87 (63)	54 (73)	30 (51)	*p* = 0.009
Not meeting guidelines	52 (37)	20 (27)	29 (49)	
Time spent sitting on a weekday hrs (*n* = 90) M ± SD				
Total weekday	7.7 ± 3.6	7.7 ± 4.2	8.0 ± 2.9	
Travel weekday	1.8 ± 3.5	1.8 ± 4.2	1.8 ± 2.5	
Work weekday	1.0 ± 1.9	1.6 ± 4.2	0.2 ± 0.6	*t*(108) = −3.99, *p* < 0.001
TV weekday	3.6 ± 3.3	3.2 ± 3.2	4.0 ± 3.4	
Home computer use weekday	1.3 ± 3.1	1.3 ± 1.7	1.3 ± 4.4	
Leisure weekday	1.7 ± 1.9	1.6 ± 1.7	2.3 ± 2.0	*t*(113) = 3.17, *p* = 0.002
Time spent sitting on a weekend day hrs (*n* = 90) M ± SD				
Total weekend day	8.0 ± 3.6	7.8 ± 4.0	8.4 ± 3.1	
Travel weekend day	1.3 ± 1.6	1.0 ± 1.1	1.6 ± 2.1	
Work weekend day	0.2 ± 0.9	0.4 ± 1.2	0.1 ± 0.2	*t*(143) = −2.72, *p* = 0.007
TV weekend day	4.0 ± 2.1	3.8 ± 2.1	4.2 ± 2.0	
Home computer use weekend day	1.1 ± 1.8	1.3 ± 1.9	0.9 ± 1.7	
Leisure weekend day	1.7 ± 1.5	1.6 ± 1.7	2.0 ± 1.3	
Objective activity data (*n* = 29)				
% wear time in each activity category				
Sedentary	67.3 ± 9.7	60.2 ± 8.2	72.3 ± 7.2	*t*(27) = 0.48, *p* < 0.001
Light	30.6 ± 8.3	36.3 ± 7.0	26.6 ± 6.6	*t*(27) = 0.10, *p* = 0.001
Moderate to vigorous	2.1 ± 2.0	3.6 ± 2.1	1.1 ± 1.1	*t*(27) = 5.06, *p* = 0.001
Time spent in each activity category hrs./day M ± SD				
Sedentary	9.6 ± 1.7	8.5 ± 1.1	10.3 ± 1.6	*t*(27) = 2.58, *p* = 0.004
Light	4.3 ± 1.2	5.2 ± 1.1	3.8 ± 1.0	*t*(27) = 1.42, *p* = 0.001
Moderate to vigorous (mins./day)	18.1 ± 17.3	30.4 ± 18.6	9.4 ± 9.7	*t*(27) = 5.97, *p* < 0.000

For self-reported sitting, a mean of almost 8 h was spent sitting on both weekdays and weekend days in the total sample (7.7 ± 3.6 and 8.0 ± 3.6 h respectively). Time spent sitting watching TV accounted for the highest amount of sitting time on a weekday (3.6 ± 3.3 h) and on a weekend day (4.0 ± 2.1 h). There were no differences between younger and older women for total sitting, travel, or home computer use on a weekday. Significant differences were found between younger and older women for work [*t*(108) = −3.99, *p* < 0.000] and leisure time sitting [*t*(113) = 3.17, *p* = 0.002], with older women spending less time sitting at work and more time sitting for leisure than younger women. At the weekend, there was a significant difference between sitting for work time only, with younger women spending more time in this domain than older women [*t*(143) = −2.72, *p* = 0.007].

### Objective physical activity and sedentary behaviour

Objective physical activity and sitting time are also reported in Table 2. The mean number of valid wear days was 5.7 ± 1.4 days, with an average of 14.2 ± 1.2 h of wear time per day. Time spent in sedentary behaviour whilst playing bingo was misclassified as non-wear time in five women’s accelerometer data totalling 20.5 h. There were no differences in the number of valid wear days or wear time per day between younger and older women.

On average, 18.1 ± 17.3 min per day was spent in moderate to vigorous physical activity (2.1% of wear time). Older women spent significantly less time in light activity per day than younger women (1.4 h less; [*t*(27) = 0.10, *p* = 0.001]) and in moderate to vigorous physical activity (21.0 min less; *t*(27) = 5.97, *p* < 0.000). Older women accumulated 9.4 ± 9.7 min of moderate to vigorous physical activity per day with younger women accumulating 30.4 ± 18.6 min per day.

For all women, most wear time was spent sedentary (9.6 ± 1.7 h per day). Significant differences were found between younger and older women for sedentary time. On average, older women spent 1.8 h longer than younger women in sedentary activities per day [*t*(27) = 0.48, *p* < 0.000], with older women accumulating 10.3 ± 1.6 h of sedentary time per day compared with 8.5 ± 1.1 in younger women.

## Discussion

The aims of this paper were to describe the socio-demographic characteristics of bingo players and to assess their health status, physical activity and sedentary behaviour. The results suggest that bingo clubs are a setting with a high proportion of women from areas of high deprivation and that future interventions should focus on increasing physical activity and reducing sedentary behaviour in older women specifically.

Physical activity interventions have been criticised in the past for attracting tertiary educated, middle class participants [17]. However, the findings of this study suggest that women from areas of high deprivation, many with no formal qualification, are well-represented in this setting, with over half of questionnaire respondents coming from the two highest quintiles of deprivation. With these women already attending the bingo club and older women unlikely to be engaged in settings such as schools and workplaces that are commonly used for the delivery of health promotion interventions, the bingo club could be an ideal novel setting to engage them in a health intervention.

This study has also provided important data on negative health behaviours to inform the development of a health intervention in a bingo club. Despite many participants coming from areas of high deprivation, self-reported physical activity levels of this group were relatively high, with nearly two-thirds saying they were already meeting physical activity guidelines. However, there was a significant difference between older and younger women with only around 50% of older women meeting the guidelines. This accords with self-reported Scottish national data which suggest that only 53% of 55 to 74 year olds are meeting guidelines [7].

As with self-reported physical activity, accelerometer-measured moderate to vigorous physical activity was also significantly lower in older women than younger women (21 min per day), with older participants not even reaching 10 min per day. Whilst this level of activity was comparable to similar age groups reported elsewhere in the literature for older women, younger women in this study achieved higher levels of moderate to vigorous physical activity by almost 10 min per day [15]. The high levels of activity reported in younger women in this sample could be explained by self-selection bias, with younger women who already have an interest in physical activity more likely to take part in a health related study. Interventions that are designed specifically to target older women in bingo clubs may be a more promising avenue for future research than targeting the whole population.

Older women also reported spending more time sitting than younger women. Whilst both age groups spent most of their sedentary time watching television, younger women, as might be expected, spent more time sitting at work on both weekdays and weekends. Accelerometer-measured sedentary time was also significantly higher in older women, with sedentary time accounting for 72% of their wear time compared to 60% in younger women. This equates to over 10 h of sedentary time per day, which is higher than that reported in other studies of older adults [18, 19]. With sedentary time associated with ill-health, even after accounting for time spent in moderate to vigorous physical activity, this is an important challenge for future interventions [20, 21].

A limitation of this study was that it took place in a single bingo club in central Scotland. Furthermore, this was a convenience sample, with bingo players self-selecting to fill in and return the questionnaire, and an even smaller self-selected sample wearing accelerometers. Although the recruitment strategy used was comprehensive and the study sample likely to be representative of this particular bingo club, it cannot be determined whether the sample was representative of bingo club members in other areas of Scotland and the UK. In addition, although this sample is not ethnically diverse (97% white Scottish), it is comparable to the general population in Scotland where 96% of people are from a white background [22]. However, this figure may differ significantly depending on where bingo clubs are based. Despite its limitations, the study does provide a strong rationale for designing a physical activity intervention for older rather than younger women in this setting. With around half of older women in the present study not meeting physical activity guidelines and spending around 10 h sitting per day, there is significant potential to influence these behaviours in this key group. However, any intervention must take into account and cater for the physical ability of this group, with a high frequency of conditions reported such as arthritis and joint problems, which may have implications for physical activity interventions.

## Conclusion

The findings of this study suggest that bingo clubs are settings that attract women from areas of high deprivation and older women in bingo clubs in particular would benefit from interventions to target their physical activity and sedentary behaviour. Bingo clubs may therefore be potential intervention settings in which to influence these behaviours.

## Abbreviations

SD: Standard deviation
SIMD: Scottish index of multiple depravation

## References

[1] Warburton DE, Nicol CW, Bredin SS (2006). Health benefits of physical activity: the evidence. CMAJ.

[2] Lee IM, Skerrett PJ (2001). Physical activity and all-cause mortality: what is the dose–response relation?. Med Sci Sports Exerc.

[3] Mammen G, Faulkner G (2013). Physical activity and the prevention of depression: a systematic review of prospective studies. Am J Prev Med.

[4] WHO. Global Strategy on Diet, Physical Activity and Health 2004 [12th April 2016]. Available from: http://www.who.int/dietphysicalactivity/strategy/eb11344/strategy_english_web.pdf. Accessed 27 July 2017.

[5] Oldridge NB (2008). Economic burden of physical inactivity: healthcare costs associated with cardiovascular disease. Eur J Cardiovasc Prev Rehabil.

[6] Scarborough P, Bhatnagar P, Wickramasinghe KK, Allender S, Foster C, Rayner M (2011). The economic burden of ill health due to diet, physical inactivity, smoking, alcohol and obesity in the UK: an update to 2006–07 NHS costs. J Public Health (Oxf).

[7] The Scottish Government. The Scottish Health Survey 2012 [12th April 2016]. Available from: http://www.gov.scot/Resource/0043/00434590.pdf. Accessed 27 July 2017.

[8] The Scottish Government (2012). The Scottish health survey physical activity topic report.

[9] Hotchkiss JW, Davies C, Gray L, Bromley C, Capewell S, Leyland AH (2011). Trends in adult cardiovascular disease risk factors and their socio-economic patterning in the Scottish population 1995–2008: cross-sectional surveys. BMJ Open.

[10] The scottish Government. Scottish Index of Multipul Deprivation [12th April 2016]. Available from: http://www.gov.scot/Topics/Statistics/SIMD. Accessed 27 July 2017.

[11] Booth ML, Owen N, Bauman A, Gore C (1996). Relationship between a 14-day recall measure of leisure-time physical activity and a submaximal test of physical work capacity in a population sample of Australian adults. Res Q Exerc Sport.

[12] The Australian Institute of Health and Wellbeing. The active Australia survey: a guide and manual for implementation, analysis and reporting. Canberra; 2003. http://www.aihw.gov.au/publication-detail/?id=6442467449. Accessed 27 July 2017.

[13] Marshall AL, Miller YD, Burton NW, Brown WJ (2010). Measuring total and domain-specific sitting: a study of reliability and validity. Med Sci Sports Exerc.

[14] Choi L, Liu Z, Matthews CE, Buchowski MS (2011). Validation of accelerometer wear and nonwear time classification algorithm. Med Sci Sports Exerc.

[15] Troiano RP, Berrigan D, Dodd KW, Masse LC, Tilert T, McDowell M (2008). Physical activity in the United States measured by accelerometer. Med Sci Sports Exerc.

[16] World Health Organization. Definition of an older or elderly person [12th April 2016]. Available from: http://www.who.int/healthinfo/survey/ageingdefnolder/en/. Accessed 27 July 2017.

[17] Waters LA, Galichet B, Owen N, Eakin E (2011). Who participates in physical activity intervention trials?. J Phys Act Health.

[18] Stamatakis E, Davis M, Stathi A, Hamer M (2012). Associations between multiple indicators of objectively-measured and self-reported sedentary behaviour and cardiometabolic risk in older adults. Prev Med.

[19] Evenson KR, Buchner DM, Morland KB (2012). Objective measurement of physical activity and sedentary behavior among US adults aged 60 years or older. Prev Chronic Dis.

[20] Matthews CE, George SM, Moore SC, Bowles HR, Blair A, Park Y (2012). Amount of time spent in sedentary behaviors and cause-specific mortality in US adults. Am J Clin Nutr.

[21] Whitfield G, Pettee Gabriel KK, Kohl HW (2014). Sedentary and active: self-reported sitting time among Marathon and half-Marathon participants. J Phys Act Health.

[22] The Scottish Government. Scotland's People. Annual Report: Results from 2012 Scottish Household Survey. 2012 [12th April 2016]. Available from: http://www.gov.scot/Resource/0043/00432400.pdf. Accessed 27 July 2017.

